# Physiological and psychological effects of walking on young males in urban parks in winter

**DOI:** 10.1186/1880-6805-32-18

**Published:** 2013-10-29

**Authors:** Chorong Song, Dawou Joung, Harumi Ikei, Miho Igarashi, Mariko Aga, Bum-Jin Park, Masayuki Miwa, Michiko Takagaki, Yoshifumi Miyazaki

**Affiliations:** 1 Center for Environment, Health and Field Sciences, Chiba University, Chiba, Japan; 2 Department of Environment and Forest Resources, Chungnam National University, Daejeon, Korea

**Keywords:** Urban green space, Walking, Winter, Stress, Physiological relaxation, Heart rate, Heart rate variability, Semantic differential method, Profile of mood states, State-trait anxiety inventory

## Abstract

**Background:**

Interaction with nature has a relaxing effect on humans. Increasing attention has been focused on the therapeutic effects of urban green space; however, there is a lack of evidence-based field research. This study provided scientific evidence supporting the physiological and psychological effects of walking on young males in urban parks in winter.

**Findings:**

Subjects (13 males aged 22.5 ± 3.1 years) were instructed to walk predetermined 15-minute courses in an urban park (test) and in the city area (control). Heart rate and heart rate variability (HRV) were measured to assess physiological responses. The semantic differential (SD) method, Profile of Mood States (POMS), and State-Trait Anxiety Inventory (STAI) were used to determine psychological responses.

Heart rate was significantly lower and the natural logarithm of the high frequency component of HRV was significantly higher when walking through the urban park than through the city area. The results of three questionnaires indicated that walking in the urban park improved mood and decreased negative feelings and anxiety.

**Conclusions:**

Physiological and psychological data from this field experiment provide important scientific evidence regarding the health benefits of walking in an urban park. The results support the premise that walking in an urban park has relaxing effects even in winter.

## Findings

### Background

It is empirically known that interaction with nature has a relaxing effect. As the interest in health promotion and quality of life has increased, attention has been focused on the role of nature in promoting human health and well-being.

Humans evolved into what they are today after the passage of six or seven million years [1]. Therefore, more than 99.99% of human evolutionary history was spent in the natural environment. Urbanization can be defined as a post-industrial revolutionary development. Through centuries of evolution within the natural environment, humans adapted to nature [2,3]. This human tendency to be close to nature implies that contact with nature may be an important component of well-being [4].

Many studies have demonstrated significant positive psychological and physiological benefits of interaction with nature. Interaction with nature aids recovery after attentional fatigue [5] and stress [2] and improves emotional state [6]. Studies of the physiological effects of relaxation in forest environments have tested parameters such as cerebral activity in the prefrontal area [7], pulse rate [8-10], blood pressure [9,11], heart rate variability (HRV) [8,10,11], salivary cortisol concentration [7-10], and natural killer (NK) cell activity [12,13]. Many results based on scientific evidence have been reported [14-16].

However, in modern society, interaction with nature such as forests is difficult. Recently, increasing attention has been focused on the role of urban green spaces such as urban parks that provide a natural environment close to most people in modern society. Recent demographic research found a positive association between exposure to urban green space and perceived general health of residents [17]. Living in areas with walkable green spaces positively influenced the longevity of urban senior citizens independent of their age, sex, marital status, baseline functional status, and socioeconomic status [18].

However, there is a lack of evidence-based research on the therapeutic effects of urban green space. Many scientists have emphasized the importance of field research [19]. Because the therapeutic effects of nature occur without conscious thought, these effects must be clarified in physiological field studies [3]. Furthermore, although we conducted similar experiments during summer [20], there are no experimental examples that can verify the physiological effects during winter.

The aim of this study was to provide scientific evidence supporting the physiological and psychological effects of walking in urban parks in winter.

## Methods

**Figure 1:**
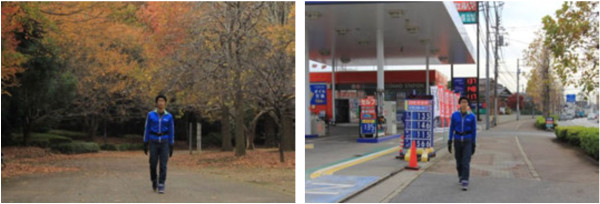
Experimental scene of urban park (left) and city area (right).

The field experiment was performed in November 2012 in Kashiwanoha Park (hereinafter referred to as the urban park) in Chiba, Japan. As a control, the city area around the urban park (hereinafter referred to as the city area) was selected (Figure 1). The weather on the day of the experiment was sunny, and the average temperature, humidity, and intensity of illumination in the urban park were 13.8°C, 50.9%, and 7,930 lx, respectively, while those in the city area were 14.0°C, 52.1%, and 8,430 lx, respectively. In addition, the trees in the park had either lost their leaves or the leaves had turned red or yellow, but there was no snow.

Thirteen Japanese male university students (22.5 ± 3.1 years old) participated in this experiment. Each subject walked in the urban park or city area for 15 minutes. None reported a history of physical or psychiatric disorders. This study was performed according to the regulations of the Ethics Committee of the Center for Environment, Health and Field Sciences, Chiba University, Japan.

Heart rate and HRV were measured to assess physiological responses. HRV, which is often used to assess human autonomic activity, was measured using a portable electrocardiograph (Activtracer AC-301A, GMS, Tokyo, Japan). HRV data were obtained at various frequencies using an HRV software tool (MemCalc/Win, GMS). For real-time HRV analysis using the maximum entropy method, inter-beat (R-R) intervals were obtained continuously. In this study, the two major HRV spectral components, low frequency (LF; 0.04 to 0.15 Hz) and high frequency (HF; 0.15 to 0.40 Hz) band variance, were calculated. The LF/HF ratio in R-R interval variability was also assessed. HF components can be a general indication of parasympathetic nervous activity, and the LF/HF ratio can be used as an index of sympathetic nervous activity [21,22]. To normalize the distribution of HRV parameters, we used natural logarithmic transformed values for the analysis [23]. The heart rate and HRV data, which were collected at 1 minute intervals at each experimental location, were compared based on the average for 15 minutes.

Three different questionnaires were used to investigate psychological responses. The questionnaires were completed after walking at each experimental site. Evaluation using semantic differential (SD) method [24] was performed using three pairs of adjectives on seven scales, including ‘comfortable to uncomfortable’, ‘natural to artificial’, and ‘relaxed to awakening’. The Profile of Mood State (POMS) data were analyzed using the following six subscales: ‘tension–anxiety’, ‘depression’, ‘anger–hostility’, ‘vigor’, ‘fatigue’, and ‘confusion’. In the POMS test, a short form with 30 questions was used to reduce the burden on the subjects [25,26]. The State-Trait Anxiety Inventory (STAI) [27] was used to evaluate anxiety.

We performed a within-subject experiment, and to eliminate the effect of the order of sites walked, two subjects were paired. One subject walked in the urban park first and in the city area later, while the other walked in the city area first and then in the urban park. There was no difference in the physiological index before the start of each walk between the two environments. After walking, the subjects returned to the waiting room and completed questionnaires. They rested for approximately 20 minutes and repeated the experiment by walking at the alternate experimental site. In addition, there was no difference in the walking speed between the two environments.

All data were shown as mean ± standard deviation. A paired *t*-test was used to compare the differences in the mean physiological data scores over a period of 15 minutes while walking in the urban park and city area. Wilcoxon signed-rank test was used to analyze differences in the psychological indices after walking between the two environments. Statistical analysis was performed using SPSS 20.0 (SPSS Inc, Chicago, IL, USA). A one-sided test was used in this study. In all cases, values of *P* <0.05 were considered statistically significant.

## Results and discussion

**Figure 2:**
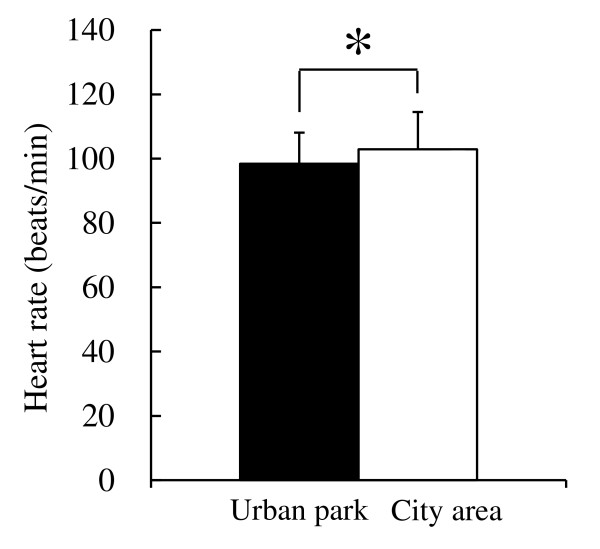
**Comparison of heart rates of subjects walking in the urban park and city area.** n = 8, mean ± SD. *P <0.05, determined by paired *t*-test.

**Figure 3:**
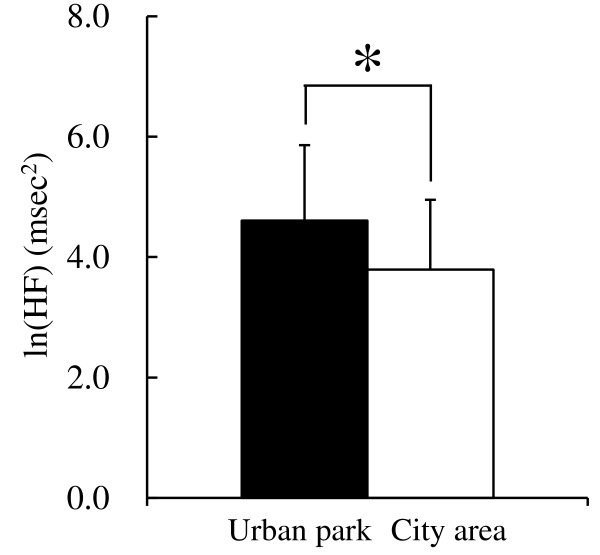
**Comparison of ln(HF) values between urban park and city area.** n = 7, mean ± SD. ln(HF), natural logarithm of high frequency. *P <0.05, determined by paired *t*-test.

In the comparison of physiological indices between the urban park and the city area, key differences were observed. Subjects’ heart rates were significantly lower (4.4%) after walking in the urban park (98.4 ± 0.9 bpm) than after walking in the city area (102.9 ± 1.1 bpm; *P* <0.05; Figure 2). When the results of HRV data were compared, a significant difference was found in the natural logarithm of HF (ln(HF)), which is a marker of parasympathetic nervous activity, between the two environmental stimuli. The urban park (4.61 ± 1.25 msec^2^) showed a 21.6% higher value than the city area (3.79 ± 1.16 msec^2^; *P* <0.05; Figure 3). In the comparison of ln(LF/HF) values, which is a marker of sympathetic nervous activity, no significant difference between the two environments was found. However, a trend towards lower values in the urban park (1.06 ± 1.01 ratio) compared with the city area (1.40 ± 1.00 ratio) was detected (*P* = 0.06). These physiological reactivity results correlated partly with those reported by previous studies related to forest therapy [8,10,11,14-16], supporting the hypothesis that urban parks have similar health benefits to natural environments.

**Figure 4:**
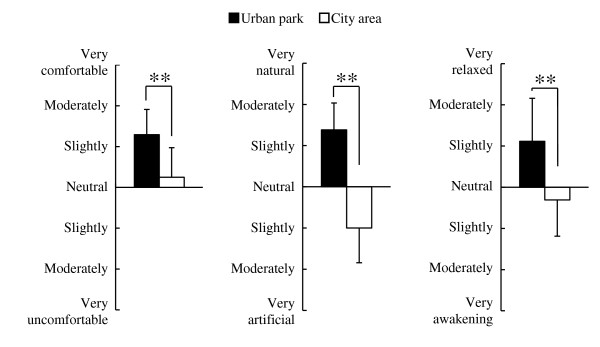
**Comparison of subjective scoring for comfortable, natural, and relaxed feelings between the two environments.** n = 13, mean ± SD. **P <0.01, determined by Wilcoxon signed-rank test.

**Figure 5:**
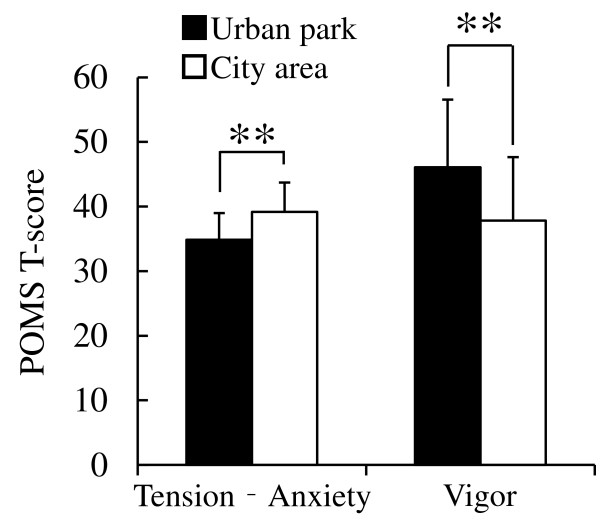
**Comparison of subjective scoring for tension–anxiety and vigor by POMS between the two environments.** n = 13, mean ± SD. POMS, Profile of Mood States. **P <0.01, determined by Wilcoxon signed-rank test.

**Figure 6:**
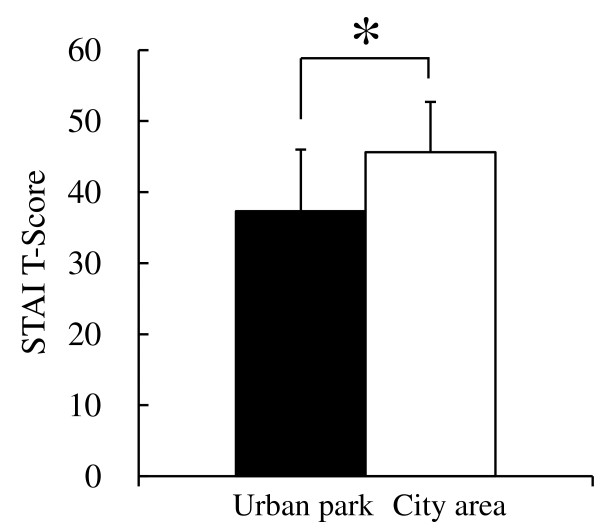
**Comparison of subjective scoring for state anxiety by STAI between the two environments.** n = 13, mean ± SD. STAI, State-Trait Anxiety Inventory. *P <0.05, determined by Wilcoxon signed-rank test.

The results of the analysis of psychological responses revealed notable differences between the two environments. In the comparison of the SD scores, significantly higher scores were observed in the urban park for the following three adjectives: ‘comfortable’, ‘natural’, and ‘relaxed’ compared with the city area (*P* <0.01; Figure 4). Significant differences were also detected in the results of the POMS test (Figure 5). The score for the negative subscale ‘tension–anxiety’ was significantly lower after walking in the urban park compared with the city area (*P* <0.01). Conversely, the positive mood state for ‘vigor’ was significantly higher in the urban park but not in the city area (*P* <0.01). For the other subscales (‘depression’, ‘anger–hostility’, ‘fatigue’, and ‘confusion’), no significant differences were observed. In the results of analysis of state anxiety using STAI, the score was 18.2% lower in the urban park (37.3 ± 8.7 scores) compared with the city area (45.6 ± 7.1 scores; *P* <0.05; Figure 6).

Based on the analysis of these three psychological indices, subjects felt more comfortable, natural, relaxed, and vigorous when walking in the urban park than in the city area. In addition, the negative emotions and anxiety levels were significantly lower. These results on the psychological benefits of walking in the urban park are partly consistent with previous findings related to forest therapy [8-11].

For those who desire a higher quality of life, scientific evidence about the relaxation effects of urban green space must be accumulated, as these are the natural environments most accessible to people in modern society.

However, these results cannot be extrapolated to the female population and people of different age groups or ethnicities, because only thirteen young male adults participated in this study. To generalize the findings, further evidence-based studies on a large sample including various subject groups is required.

This study may contribute toward progress in the area of physiological anthropology because identifying the physiological effects of the natural environment is an important issue.

## Conclusion

These findings provide important scientific evidence of the health benefits of walking in urban parks. The results support the premise that walking in urban parks has relaxing effects even in winter.

## Abbreviations

HF: High frequency; HRV: Heart rate variability; LF: Low frequency; NK: Natural killer; POMS: Profile of mood states; SD: Semantic differential; STAI: State-trait anxiety inventory.

## Competing interests

The authors declare that they have no competing interests.

## Authors’ contributions

CS contributed to the study design, data acquisition, statistical analysis, interpretation of the results, and manuscript preparation. DJ, HI, MI, and MA were involved with data acquisition and statistical analysis. BP conceived and designed the study. MM and MT participated in the design of the study and carried out interpretation of data. YM had an important role in the research, particularly in experimental design, data acquisition, and manuscript preparation. All authors contributed to the preparation and are responsible for the final editing and approval of the manuscript.
